# Canonical ETI‐Dependent and ‐Independent Pathways Mediate Autoimmunity Caused by Loss of CBP60b Clade Function

**DOI:** 10.1111/mpp.70318

**Published:** 2026-07-11

**Authors:** Lu‐Shen Li, Yan‐Yan Yang, Lin Ma, Shu‐Lei Wang, Sha Li

**Affiliations:** ^1^ College of Agriculture and Biology Liaocheng University Liaocheng China; ^2^ State Key Laboratory of Wheat Improvement, College of Life Sciences Shandong Agricultural University Tai'an China; ^3^ Frontiers Science Center for Cell Responses, College of Life Sciences Nankai University China

**Keywords:** autoimmunity, calmodulin‐binding protein 60b (CBP60b), CBP60g, effector‐triggered immunity (ETI), pathogen, transcriptional activity

## Abstract

Calmodulin‐Binding Protein 60 (CBP60), a plant‐specific atypical transcription factor family, plays pivotal roles in reprogramming plant immunity. Loss of function of the CBP60b and CBP60b/c/d/e/f subfamily constitutively activates immune responses (autoimmunity). However, the specific mechanisms and pathways underlying this autoimmunity remain elusive. Here, through EMS mutagenesis of the *cbp60b* mutant, we isolated a dominant suppressor mutant that suppresses the *cbp60b* autoimmune phenotype. Sequencing identified a point mutation in *CBP60g* (designated *cbp60g‐3*) that causes an aspartate‐to‐asparagine substitution at position 252 and reduces its transcriptional activity. Increasing the expression of *CBP60g* ^*D252N*^ restored the pathogen response defect of the *cbp60g;sard1* mutant. Strikingly, while wild‐type CBP60g failed to suppress the autoimmunity of *cbp60b*, the transcriptionally compromised CBP60g^D252N^ variant completely suppressed it. Unexpectedly, we found that functional loss of canonical effector‐triggered immunity (ETI) pathways only partially suppressed the autoimmunity of the *cbp60b;c;d;e;f* quintuple mutant. Although simultaneously disrupting the ETI pathways and introducing CBP60g^D252N^ further suppressed the autoimmunity of *cbp60b;c;d;e;f*, it still failed to achieve complete suppression. These results indicate that the autoimmunity in *cbp60b;c;d;e;f* mutant is mediated by both canonical ETI‐dependent and ETI‐independent pathways. These results provide new insights and raise further questions regarding the signalling mechanisms of plant autoimmunity.

## Graphical abstract

A CBP60g point mutant (CBP60g^D252N^) with reduced transcriptional activity rescues the autoimmunity of the *cbp60b* mutant. Introducing CBP60g^D252N^ in the *cbp60b;c;d;e;f* mutant background fails to completely suppress the autoimmunity of *cbp60b;c;d;e;f*, suggesting that canonical ETI‐dependent and ‐independent pathways mediate autoimmunity caused by loss of CBP60b clade function.

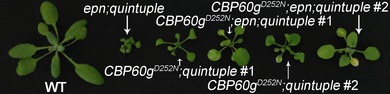

## 1 Introduction

Plants recognize pathogenic microorganisms through two primary immune pathways. Initially, pattern recognition receptors (PRRs) on the cell surface perceive pathogen‐associated molecular patterns (PAMPs) or damage‐associated molecular patterns (DAMPs) generated upon pathogen invasion, thereby activating pattern‐triggered immunity (PTI) (Zhou and Zhang 2020; Ngou et al. 2022; Zhang et al. 2023; Weralupitiya et al. 2024). To establish successful infection, pathogens secrete effectors into plant cells to enhance virulence (Jones et al. 2024). In response, intracellular immune receptors known as nucleotide‐binding leucine‐rich repeat (NLR) proteins recognize these effectors, triggering effector‐triggered immunity (ETI) (Zhou and Zhang 2020; Ngou et al. 2022; Wang et al. 2022; Huang et al. 2023). NLRs are classified into three subfamilies: TNLs, CNLs and RNLs (Zhou and Zhang 2020). The formation of enhanced disease susceptibility 1 (EDS1)‐phytoalexin deficient 4 (PAD4) or EDS1‐senescence‐associated gene 101 (SAG101) complexes and RNLs are essential for TNL‐mediated ETI signaling (Zhou and Zhang 2020; Huang et al. 2023). In contrast, CNL‐mediated immunity primarily requires non‐race‐specific disease resistance 1 (NDR1) for signal transduction (Jones and Dangl 2006; Dodds and Rathjen 2010). ETI activation often culminates in the hypersensitive response (HR), a form of localized programmed cell death that restricts pathogen spread (Dodds and Rathjen 2010; Zhou and Zhang 2020). Accumulating evidence indicates that PTI and ETI mutually potentiate each other through synergistic interactions, ensuring robust and sustained immune outputs during pathogen challenge (Yuan et al. 2021; Lozano‐Durán et al. 2023).

Certain gene mutations can aberrantly activate plant immune responses, leading to cell death and developmental abnormalities. Such mutants are referred to as autoimmune or lesion mimic mutants (Rodriguez et al. 2016; van Wersch et al. 2016; Chakraborty et al. 2018; Freh et al. 2022). Typical phenotypes of autoimmune mutants include dwarfism or semi‐dwarfism, spontaneous leaf lesions, elevated salicylic acid (SA) levels, constitutive expression of immune‐related genes, and enhanced resistance to pathogens (Chakraborty et al. 2018; Freh et al. 2022). Autoimmunity in plants can result from gain‐of‐function mutations in immune receptors or loss‐of‐function mutations in negative regulators of immunity (Chakraborty et al. 2018; Freh et al. 2022). More frequently, however, autoimmunity arises from mutations in targets of NLR‐recognized effectors (Rodriguez et al. 2016; van Wersch et al. 2016). The phenotypes of such mutants can often be suppressed by introducing mutations in specific NLRs or in key components of the ETI signalling pathway, such as EDS1 or PAD4 (Zhang et al. 2012; Rodriguez et al. 2016; Chakraborty et al. 2018; Takagi et al. 2019). The mechanism underlying this type of autoimmunity is frequently explained by the “guard model”: the mutated gene encodes a protein that is normally targeted by pathogen effectors; when this guardee is altered, the corresponding NLR perceives this modification as a sign of infection and erroneously activates immune responses (Jones and Dangl 2006; Rodriguez et al. 2016; Freh et al. 2022). In addition to autoimmunity caused by gain‐ or loss‐of‐function mutations in genes directly involved in immune signalling, loss‐of‐function mutations in biological processes seemingly unrelated to immunity, such as nitrogen metabolism and nonsense‐mediated RNA decay (NMD), can also lead to autoimmune phenotypes (Riehs‐Kearnan et al. 2012; Colinas et al. 2016).

Regardless of the underlying cause, autoimmune phenotypes can often be suppressed by introducing mutations in key components of immune signalling pathways, such as EDS1, PAD4 or NDR1 in the ETI pathway; SID2 or NPR1 in the salicylic acid (SA) pathway; FMO1 in the systemic acquired resistance (SAR) pathway; or CBP60g and SARD1 (Sun et al. 2015; van Wersch et al. 2016; Chakraborty et al. 2018). Moreover, the distinct morphological phenotypes of autoimmune mutants facilitate the visual screening of suppressors, which are of great significance for elucidating the mechanisms of plant immune receptors and dissecting their associated signalling networks (Johnson et al. 2013; van Wersch et al. 2016; Chakraborty et al. 2018).

The calmodulin‐binding protein 60 (CBP60) family comprises plant‐specific transcription factors. In *Arabidopsis thaliana* , this family consists of eight members, which are divided into four subfamilies/clade: CBP60a, CBP60b/c/d/e/f, CBP60g and SARD1 (Ding and Redkar 2018; Zheng et al. 2021; Li et al. 2024). The CBP60b clade homologous genes are present across land plant lineages, whereas the other clades evolved around the time of angiosperm diversification (Zheng et al. 2021). CBP60a functions as a negative regulator of plant immunity (Truman et al. 2013; Lu et al. 2018), although its precise molecular mechanism remains unclear (Lu et al. 2018; Zheng et al. 2021). Unlike other family members that localize exclusively to the nucleus, CBP60a is distributed in both the nucleus and cytoplasm (Li et al. 2024). CBP60g and SARD1 act redundantly to regulate the expression of immune genes involved in PTI, ETI, SA signalling and SAR (Zhang, Xu, et al. 2010; Wang et al. 2011; Sun et al. 2015; Huang et al. 2020). CBP60b is considered a key activator of immune transcriptional reprogramming. On one hand, it regulates the expression of a suite of immune‐related genes. In particular, reverse transcription‐quantitative PCR (RT‐qPCR), dual luciferase reporter assays and chromatin immunoprecipitation (ChIP) assays have demonstrated that CBP60b directly binds to the promoters of *CBP60g* and *SARD1* and activates their expression (Li et al. 2021). On the other hand, the *cbp60b* mutant exhibits autoimmunity that is dependent on canonical EDS1‐PAD4 and NDR1 ETI pathways; introducing mutations in the EDS1‐PAD4 or NDR1 pathway can completely or partly suppress the *cbp60b* autoimmune phenotype (Huang et al. 2021; Li et al. 2021). This suggests that CBP60b may be monitored as a guardee by NLR proteins. Indeed, evidence indicates that CBP60g is targeted by pathogen effectors. The effector VdSCP41 from *Verticillium dahliae* binds to the transcriptional activation domain of CBP60g, inhibiting their function (Qin et al. 2018). Furthermore, loss‐of‐function of the entire CBP60b–f clade or of CBP60b in combination with CBP60g and SARD1 results in more severe autoimmunity (Li et al. 2021, 2024). The *cbp60b* phenotype can be rescued by expressing *CBP60d* or *CBP60f* under the control of the *CBP60b* promoter, but not by expressing *CBP60g* or *SARD1*. Interestingly, both the *cbp60b;c;d;e;f* quintuple mutant and the *cbp60g;cbp60b* double mutant exhibited more severe autoimmunity than the *cbp60b* single mutant (Li et al. 2021, 2024). This indicates functional redundancy among members of the CBP60b clade and reveals both overlapping and distinct functions between CBP60g/SARD1 and CBP60b (Li et al. 2024).

Although we previously demonstrated genetically that the autoimmunity of *cbp60b* is dependent on the EDS1‐PAD4 or NDR1 pathways (Li et al. 2021), it remains unclear which other immune pathways or components are involved in this process. Here, we first generated *sid2* and *fmo1* knockout mutants in the *cbp60b/+* background using CRISPR‐Cas9. Disruption of the SA and SAR pathways did not alter the autoimmune phenotype of *cbp60b*, despite the high expression levels of genes in these pathways in the mutant. Similarly, mutating *NLR* genes that are upregulated in *cbp60b* also failed to suppress its autoimmunity. We therefore performed EMS mutagenesis on *cbp60b* and screened for morphological suppressors. This led to the identification of a point mutation in *CBP60g*, which we designated *cbp60g‐3*, that was able to suppress the *cbp60b* autoimmune phenotype. *cbp60g‐3* results in an amino acid substitution at position 252 of CBP60g. This mutation reduces its transcriptional activity on downstream genes but, importantly, retains sufficient function to complement the pathogen response defect of the *cbp60g;sard1* mutant. We found that neither introducing the CBP60g^D252N^ variant nor functional loss of the canonical ETI pathway could fully suppress the phenotype of the *cbp60b;c;d;e;f* mutant. While introducing CBP60g^D252N^ into the *eds1;pad4;ndr1;cbp60b;c;d;e;f* background led to further phenotypic suppression, complete suppression was not achieved. These results indicate that both ETI‐dependent and ETI‐independent pathways mediate the autoimmunity observed in the *cbp60b;c;d;e;f* mutant.

## 2 Results

### 2.1 The Autoimmunity of *cbp60b* Is Not Mediated by the SA/SAR Pathways or by the *NLR* Genes That Are Highly Expressed in *cbp60b*

Loss of CBP60b function results in abnormal activation of plant immunity, with upregulated immune gene expression causing developmental defects—a phenotype known as autoimmunity. To investigate which pathways mediate the autoimmunity resulting from CBP60b loss‐of‐function, we first examined several known candidate pathways, including the SA and SAR pathways (van Wersch et al. 2016; Chakraborty et al. 2018). Consistent with previous reports, SA accumulation was elevated in the *cbp60b* mutant, and the expression of key genes involved in SA and SAR signalling was upregulated (Li et al. 2021). However, mutation of *FMO1* or *SID2*, as well as overexpression of the *NahG* (Zhao et al. 2016) in the *cbp60b* background, failed to suppress its developmental defects (Figure S1).

Previous studies have shown that *NLR* expression is upregulated in certain autoimmune mutants (Liu et al. 2017). We observed increased transcript levels of 11 *NLR* genes—namely *RMG1*, *TN2*, *SOC3*, *AT5G38340*, *AT3G04220*, *AT5G46260*, *AT5G66630*, *AT1G58390*, *AT1G12210*, *AT5G18350* and *AT1G64070*—in the *cbp60b* mutant (Figure S2A). Using CRISPR‐Cas9 to mutate each of these *NLR* genes (Figure S2B–L), we found that none of the individual knockouts suppressed the *cbp60b* autoimmunity (Figure S2M,N). To rule out potential functional redundancy among these NLRs (Lolle et al. 2017), we overexpressed dominant‐negative (DN) forms of the corresponding NLRs. However, these also failed to rescue the developmental defects of *cbp60b* (Figure S2O). Collectively, these results indicate that activation of the SA/SAR pathway and upregulation of *NLR* gene expression are not causal factors driving *cbp60b* autoimmunity but rather represent secondary consequences of the autoimmune state.

### 2.2 CBP60g^D252N^ Suppresses the Autoimmunity of *cbp60b*

**FIGURE 1:**
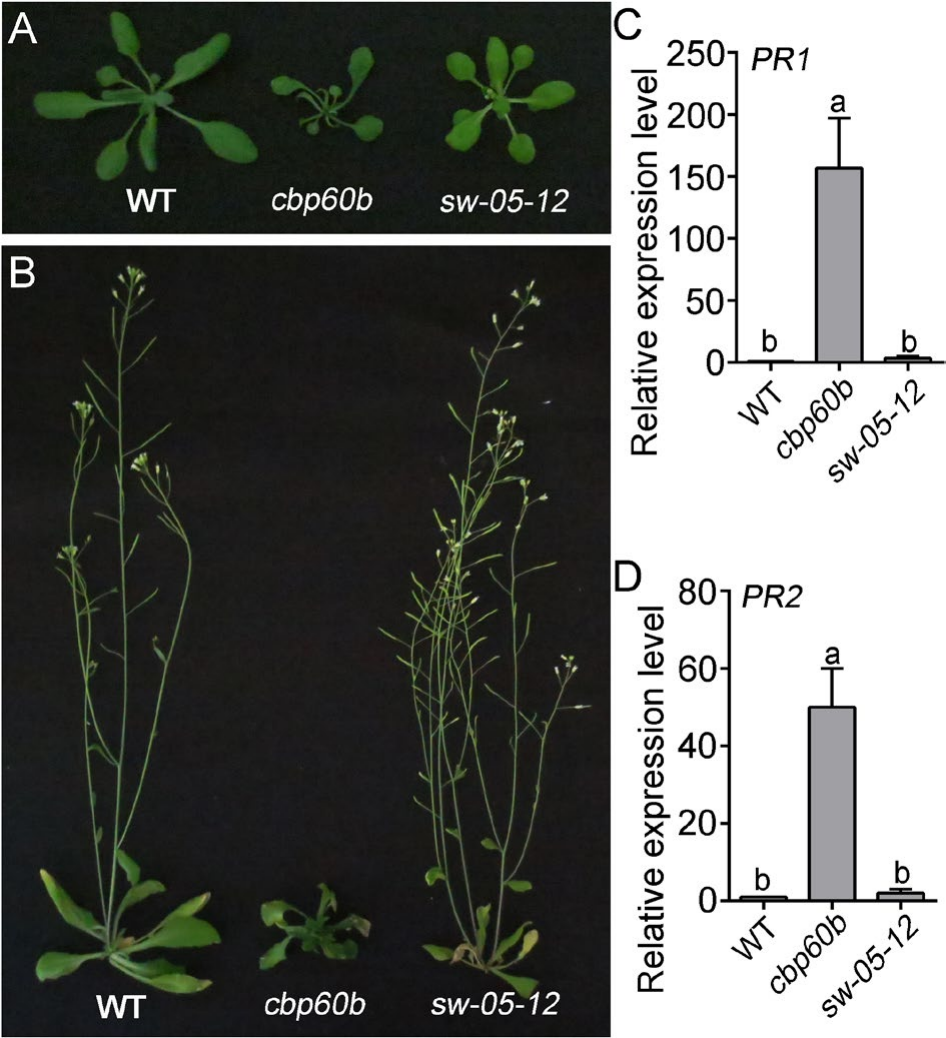
*sw‐05‐12* rescues the defects in *cbp60b*. (A, B) Representative growth of wild type (WT), *cbp60b‐1* (*cbp60b*) and *sw‐05‐12* at 3 weeks after germination (WAG) (A) or 5 WAG (B) under long‐day (LD) conditions. (C, D) Relative transcript abundance of *PR1* (C) and *PR2* (D) in the indicated genotypes. RNAs were extracted from leaves of 3 WAG plants under LD conditions. Values are means ± SE (*n* = 4). Different letters indicate significantly different groups (one‐way ANOVA and Tukey's multiple comparison test; *p* < 0.05).

To identify the immune components involved in the autoimmunity of *cbp60b*, we performed EMS mutagenesis on *cbp60b* seeds. A line with a suppressed phenotype, designated *sw‐05‐12* (Figure 1), was identified in the M_1_ generation. Approximately three‐quarters of its self‐pollinated M_2_ progeny exhibited the suppressed phenotype, indicating that it carries a dominant mutation.

**FIGURE 2:**
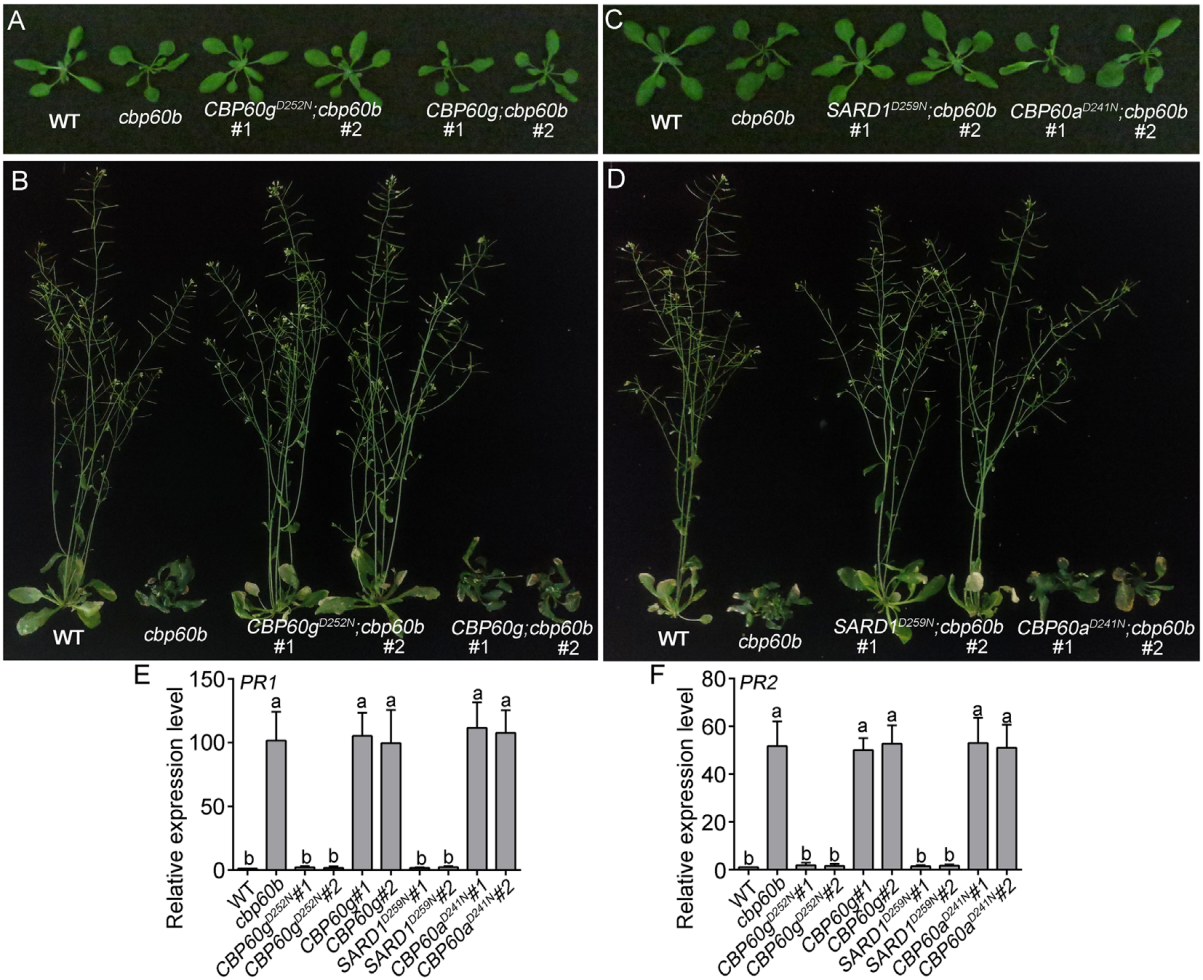
CBP60g^D252N^ and SARD1^D259N^ rescue the defects in *cbp60b*. (A, B) Representative growth of wild type (WT), *cbp60b‐2* (*cbp60b*), two independent lines of *UBQ10:CBP60g* ^*D252N*^ *‐GFP;cbp60b* and two independent lines of *UBQ10:CBP60g‐GFP;cbp60b* at 3 weeks after germination (WAG) (A) or 5 WAG (B) under long‐day (LD) conditions. (C, D) Representative growth of WT, *cbp60b*, two independent lines of *UBQ10:SARD1* ^*D259N*^ *‐GFP;cbp60b* and two independent lines of *UBQ10:CBP60a* ^*D241N*^ *‐GFP;cbp60b* at 3 WAG (C) or 5 WAG (D) under LD conditions. (E, F) Relative transcript abundance of *PR1* (E) and *PR2* (F) in the indicated genotypes. RNAs were extracted from leaves of 3 WAG plants under LD conditions. Values are means ± SE (*n* = 4). Different letters indicate significantly different groups (one‐way ANOVA and Tukey's multiple comparison test; *p* < 0.05).

Whole‐genome resequencing revealed that the mutation in *sw‐05‐12* corresponds to a G‐to‐A substitution at nucleotide position 754 in the *CBP60g* gene, resulting in an aspartic acid (D) to asparagine (N) change at amino acid 252. This allele was designated *cbp60g‐3*. CBP60g and CBP60b belong to different subfamilies within the same protein family. Our previous work demonstrated that CBP60b regulates the expression of *CBP60g* (Li et al. 2021); however, the *proCBP60b:CBP60g* failed to complement the *cbp60b* phenotype (Li et al. 2024). Furthermore, the *cbp60b;cbp60g* double mutant exacerbates the autoimmunity of *cbp60b*, suggesting partial functional redundancy between these two proteins (Li et al. 2021). To verify the mutation, we introduced the *UBQ10:CBP60g* ^*D252N*^ construct into the *cbp60b* mutant. The results showed that CBP60g^D252N^ suppressed the *cbp60b* autoimmune phenotype (Figure 2A,B,E,F).

To rule out differences in expression level and pattern, we generated and introduced *UBQ10:CBP60g* construct into *cbp60b* mutant. We found that CBP60g failed to suppress the phenotype (Figure 2A,B,E,F), which is consistent with the results driven by the *CBP60b* promoter (Li et al. 2024). These results indicate that the suppression observed in *sw‐05‐12* is mediated by the mutant CBP60g^D252N^, and that this suppression is due to the altered function of the mutant protein, rather than changes in its expression level.

The Asp252 is conserved across the *CBP60* gene family, including in early land plant lineages and higher plant lineages such as cucumber and tomato (Li et al. 2024). We have previously reported both functional similarities and differences among different CBP60 subclades at the genetic level (Li et al. 2024). To investigate the function of this residue in other clades, we introduced *UBQ10:SARD1* ^*D259N*^ and *UBQ10:CBP60a* ^*D241N*^ constructs into the *cbp60b* background. We found that SARD1^D259N^ rescued the phenotype, whereas CBP60a^D241N^ did not (Figure 2C–F). Together, these results further demonstrate that CBP60g and SARD1 are functionally similar, while CBP60a is functionally distinct from other CBP60 clades (Li et al. 2024).

To further clarify the impact of endogenous *CBP60g* transcripts on the phenotype, we designed and used specific primers to detect endogenous, exogenous, and total *CBP60g* transcripts in wild type, *cbp60b*, *UBQ10:CBP60* ^*D252N*^, *UBQ10:CBP60g*, and *UBQ10:SARD1* ^*D259N*^ in *cbp60b* transgenic lines. As previously reported (Li et al. 2021), endogenous *CBP60g* transcripts were constitutively highly expressed in *cbp60b* (Figure S3). In the *UBQ10:CBP60* ^*D252N*^ and *UBQ10:SARD1* ^*D259N*^ transgenic lines, endogenous *CBP60g* expression was suppressed, whereas in the *UBQ10:CBP60g* transgenic lines it was not suppressed and remained at levels similar to those in *cbp60b* (Figure S3). Total *CBP60g* transcript levels in the *UBQ10:CBP60* ^*D252N*^ transgenic lines were comparable to exogenous *CBP60g* transcripts, but in the *UBQ10:CBP60g* transgenic lines they were higher than exogenous *CBP60g* transcripts (Figure S3). These results indicate that the complementation of *cbp60b* by *UBQ10:CBP60* ^*D252N*^ and *UBQ10:SARD1* ^*D259N*^ is unrelated to endogenous *CBP60g* transcripts.

### 2.3 CBP60g^D252N^ Exhibits Reduced Transcriptional Activity

**FIGURE 3:**
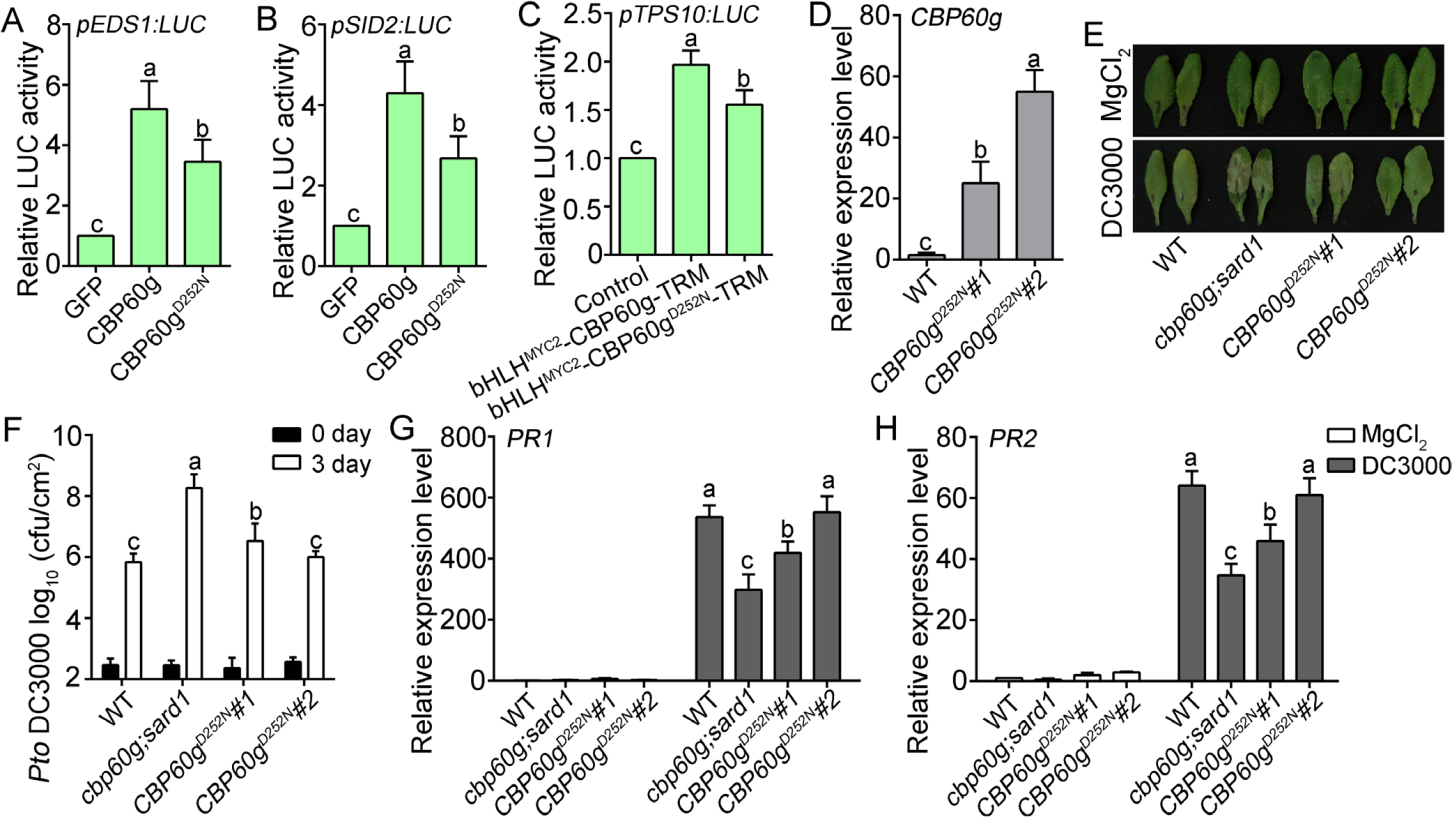
The transcriptional activity of CBP60g^D252N^ is reduced. (A, B) Quantitative luminescence showing the luciferase (LUC) activity of *pEDS1:LUC* (A) and *pSID2:LUC* (B). The *promoter:LUC* constructs were co‐transfected with *UBQ10:GFP* (GFP), *UBQ10:CBP60g‐GFP* (CBP60g), or *UBQ10:CBP60g* ^*D252N*^ *‐GFP* (CBP60g^D252N^). (C) Quantitative luminescence. *pTPS10:LUC* was transfected with *35S:BHLH* ^*MYC2*^ *‐GFP*(Control), *35S:BHLH* ^*MYC2*^ *‐CBP60g‐TRM‐GFP* (bHLH^MYC2^‐CBP60g‐TRM), or *35S:BHLH* ^*MYC2*^ *‐CBP60g* ^*D252N*^ *‐TRM‐GFP* (bHLH^MYC2^‐CBP60g^D252N^‐TRM). LUC reporter activity was determined 16 h post‐transfection. Values are means ± SE (*n* = 4). (D) Relative transcript abundance of *CBP60g* in wild‐type (WT) and two independent lines of *UBQ10:CBP60g* ^*D252N*^ *‐GFP* by reverse transcription‐quantitative PCR. (E) Representative leaf symptom photographs at 3 days post‐inoculation (dpi) with *Pseudomonas syringae* pv. *tomato* (Pto) DC3000 in the indicated genotypes. (F) Sensitivity of the indicated genotypes to Pto DC3000. Bacterial growth was determined 2 h post‐inoculation (0 d) or 3 days post‐inoculation (3 d). Values are means ± SD (*n* = 5). Experiment was repeated three times with similar results. (G, H) Relative transcript abundance of *PR1* (G), and *PR2* (H) upon mock treatment (MgCl_2_) or Pto DC3000 treatment in designated genotypes. RNAs were extracted from infiltrated leaves at 24 h post‐treatment. Results are means ± SE (*n* = 3). Different letters indicate significantly different groups (one‐way ANOVA and Tukey's multiple comparison test; *p* < 0.05).

The Asp252 of the CBP60g is located within the transcriptional regulatory motif (TRM) (Qin et al. 2018; Li et al. 2021). To investigate the impact of this residue on protein function, we performed dual luciferase (LUC) reporter assays. We co‐transfected reporter constructs *proSID2:LUC* or *proEDS1:LUC*, with *UBQ10:GFP*, *UBQ10:CBP60g‐GFP*, or *UBQ10:CBP60g* ^*D252N*^ *‐GFP* into *Arabidopsis* mesophyll protoplasts and measured relative LUC activity. We found that the LUC activity induced by CBP60g^D252N^ was significantly lower than that induced by CBP60g (Figure 3A,B), indicating that mutation of this residue is critical for its activity as a transcription factor. To further validate this result, we tested the activation capacity of the TRM (Qin et al. 2018; Li et al. 2021) from CBP60g^D252N^. The results showed that CBP60g^D252N^‐TRM exhibited significantly lower activation compared to CBP60g‐TRM (Figure 3C). Together, these results demonstrate that the Asp252 of CBP60g is essential for its function.

To further investigate the impact of this residue on protein function, we introduced the *UBQ10:CBP60g* ^*D252N*^ construct into the *cbp60g;sard1* mutant. We selected two lines with different expression levels (Figure 3D) and performed pathogen infection assays to assess whether they could complement the immune phenotype of the double mutant. We found that the extent of phenotypic rescue by CBP60g^D252N^ correlated with its expression level: high‐expressing CBP60g^D252N^ fully restored resistance to pathogen *Pseudomonas syringae* pv. *tomato* DC3000 in the double mutant, whereas low‐expressing CBP60g^D252N^ only partially restored the phenotype (Figure 3E–H). This suggests that although the point‐mutated CBP60g^D252N^ exhibits reduced transcriptional activity, its function may be compensated by increased protein abundance.

Previous studies have shown that the transcriptional activity of CBP60g is lower than that of CBP60b (Li et al. 2021), and that of CBP60g^D252N^ is lower than that of CBP60g. Therefore, the rescue of the *cbp60b* phenotype by CBP60g^D252N^ is likely not due to CBP60g^D252N^ substituting for CBP60b in transcriptional function.

Members of the CBP60b–f clade are functionally redundant, and mutations in CBP60c–f exacerbate the autoimmune phenotype of *cbp60b* (Li et al. 2024). To investigate whether CBP60g^D252N^ can suppress the autoimmunity of the *cbp60b;c;d;e;f* (*quintuple*) mutant, we introduced CBP60g^D252N^ into the *quintuple* mutant. Unexpectedly, we found that it only partially restored the *quintuple* phenotype (Figure S4).

### 2.4 The Autoimmunity of *cbp60b;c;d;e;f* Is Partially Dependent on the Canonical ETI Pathway

**FIGURE 4:**
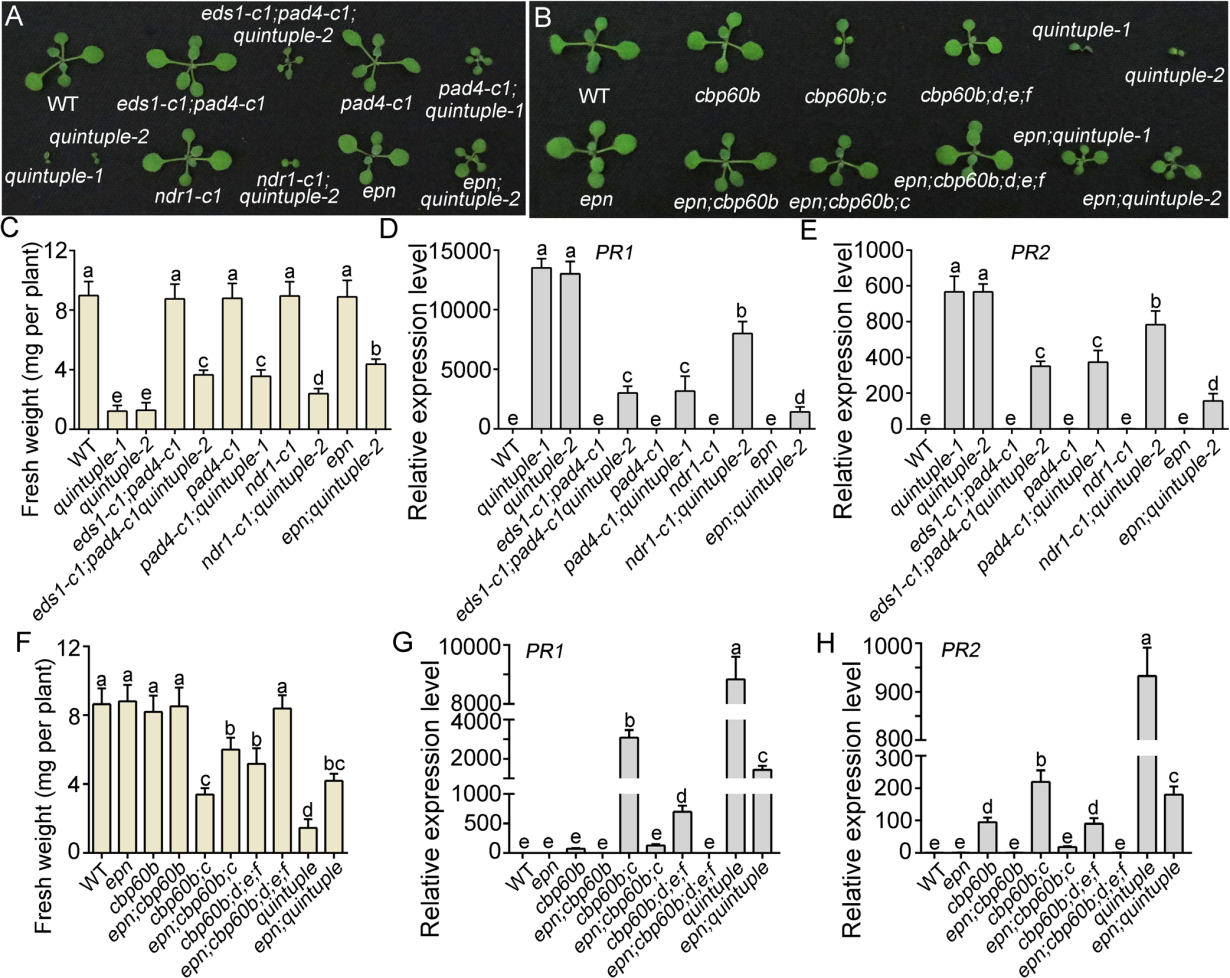
Functional loss of classical effector‐triggered immunity (ETI) pathways only partially rescue the autoimmunity in *quintuple*. (A) Representative growth of wild‐type (WT), *quintuple‐1*, *quintuple‐2*, *eds1‐c1;pad4‐c1*, *eds1‐c1;pad4‐c1;quintuple‐2, pad4‐c1*, *pad4‐c1;quintuple‐1, ndr1‐c1*, *ndr1‐c1;quintuple‐2*, *eds1‐c1;pad4‐c1;ndr1‐c1* (*epn*), and *epn;quintuple‐2* at 2 weeks after germination (WAG) under long‐day (LD) condition. (B) Representative growth of WT, *cbp60b‐1* (*cbp60b*), *cbp60b;c*, *cbp60b;d;e;f*, *quintuple‐1*, *quintuple‐2*, *epn*, *epn;cbp60b*, *epn;cbp60b;c*, *epn;cbp60b;d;e;f*, *epn;quintuple‐1*, and *epn;quintuple‐2* at 2 WAG under LD condition. (C, F) Fresh weight of the indicated genotypes. Values are means ± standard deviation (SD, *n* > 15). Aerial tissues were collected from 2 WAG plants under LD condition. (D, E, G, H) Relative transcript abundance of *PR1* (D, G) and *PR2* (E, H) in the indicated genotypes. RNAs were extracted from leaves of 2 WAG plants under LD condition. Values are means ± SE (*n* = 4). Different letters in (C–H) indicate significantly different groups (one‐way ANOVA, Tukey's multiple comparisons test, *p* < 0.05).

The observation that CBP60g^D252N^ completely suppresses *cbp60b* autoimmunity but only partially suppresses autoimmunity in the *quintuple* mutant prompted us to investigate the contribution of the canonical ETI pathway to the *quintuple* autoimmune phenotype. Our previous studies showed that introducing mutations in the EDS1‐PAD4‐ADR1s pathway, which mediates TNL immune signalling, or introducing mutations in the NDR1 pathway, which mediates CNL immune signalling, into the *cbp60b* background could fully or partially suppress the autoimmune phenotype of *cbp60b* (Li et al. 2021). To explore whether CBP60g^D252N^ is associated with distinct NLR pathways, we next examined the impact of different ETI pathways on autoimmunity in the *quintuple* mutant. We introduced the *eds1‐c1;pad4‐c1* or *ndr1‐c1* mutants into *quintuple‐1* or *quintuple‐2*. We found that *eds1‐c1;pad4‐c1*, *pad4‐c1*, or *ndr1‐c1* each rescued the developmental phenotype of the *quintuple* mutant to varying degrees, but none achieved complete suppression (Figure 4A,C–E). Unexpectedly, even after simultaneously disrupting both canonical ETI responses by mutating *EDS1*, *PAD4* and *NDR1*, the autoimmune phenotype of the *eds1‐c1;pad4‐c1;ndr1‐c1;quintuple* (*epn;quintuple*) mutant remained incompletely suppressed (Figure 4A,C–E). The *epn;quintuple* plants exhibited developmental arrest around 3 weeks of age and failed to bolt or set seeds (Figure S5). Moreover, the expression levels of immune marker genes in the *epn;quintuple* mutant were substantially higher than those in *cbp60b* or *cbp60b;d;e;f* mutants (Figure 4G,H). These results indicate that the immune responses in the *epn;quintuple* mutant are not fully suppressed.

We found that different combinations of *CBP60c–f* mutations enhanced the autoimmunity of *cbp60b* to varying degrees (Figure 4B,F). The autoimmunity of *cbp60b;c* was stronger than that of *cbp60b;d;e;f* (Figure 4B,F–H), potentially due to the higher expression of *CBP60c* in aerial tissues during the seedling stage, whereas *CBP60d/e/f* expression is relatively low (Figure S6). We assessed the suppression of *cbp60b*, *cbp60b;c*, *cbp60b;d;e;f*, and the *quintuple* mutant by *epn* (Figure 4B,F–H). The *epn* mutant fully rescued the phenotypes of *cbp60b* and *cbp60b;d;e;f*. The *epn;cbp60b;c* plants developed normally, although they were slightly smaller overall than wild‐type plants (Figure 4B,F).

### 2.5 RNA‐Seq Data Reveal Constitutive Immune Activation in *epn;quinple*

**FIGURE 5:**
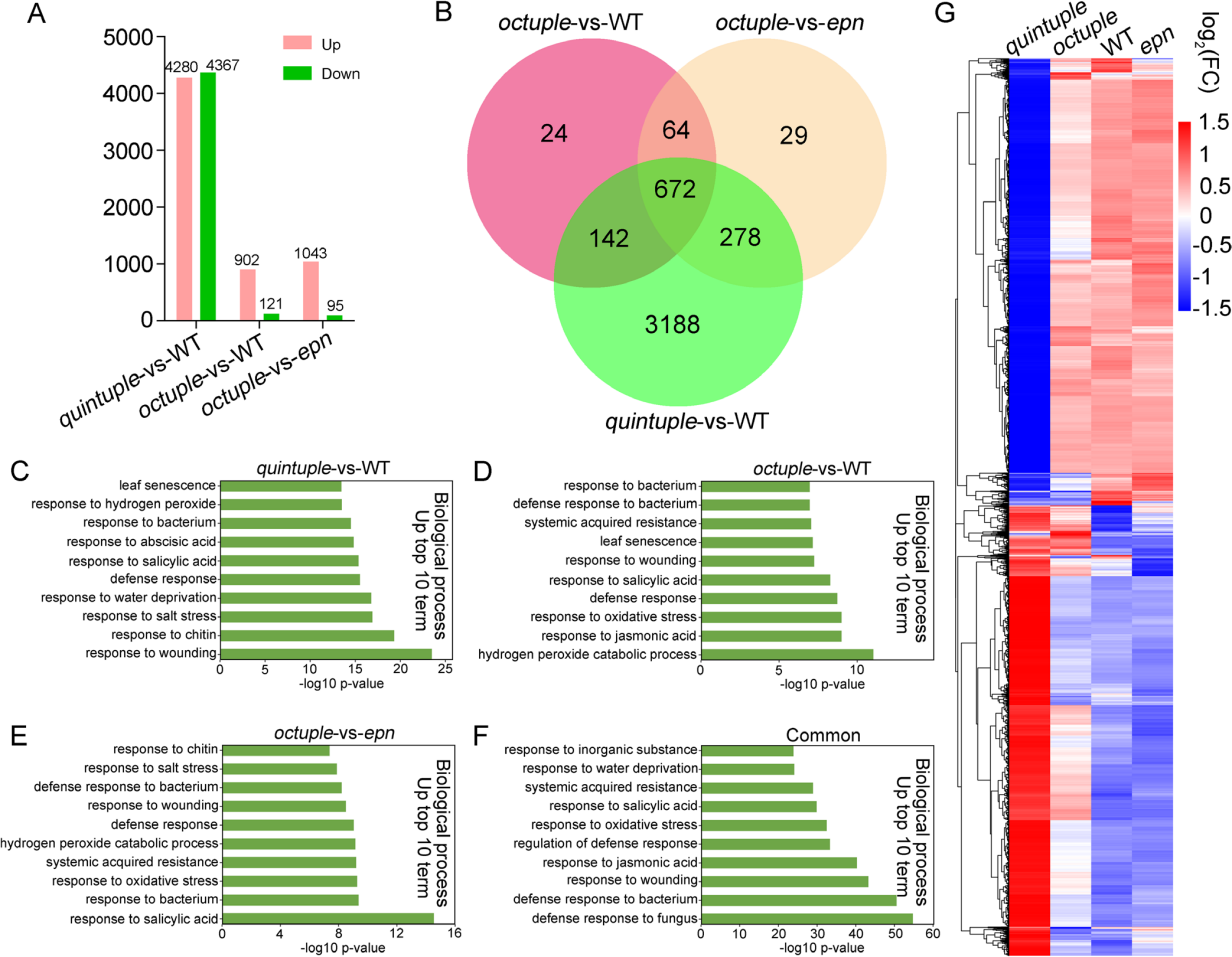
The expression of immune genes in *epn;quintuple* is elevated. (A) Statistics of differentially expressed genes (DEGs, i.e., exceed the thresholds of fold change [FC] ≥ 2.0, *q* < 0.05) in *quintuple*‐vs‐WT, *epn;quintuple* (octuple)‐vs‐WT and *octuple*‐vs‐*epn*. (B) Venn diagram shows the overlapping numbers of up‐DEGs in indicated groups. (C–F) Gene ontology enrichment of up‐DEGs in *quintuple*‐vs‐WT (C), *octuple*‐vs‐WT (D), *octuple*‐vs‐*epn* (E), and common up‐DEGs (F). The lengths of the bars indicate the −log_10_‐transformed *p*‐values. (G) Heat map of up‐ and down‐DEGs with their log_2_(FC) values.

To further investigate whether the *epn;quintuple* (*octuple*) mutant retains high immune gene expression (Figure 4), we performed RNA‐seq on aerial tissues of wild‐type (WT), *epn*, *quintuple* and *octuple* seedlings. In the comparisons *quintuple*‐vs‐WT, *octuple*‐vs‐WT and *octuple*‐vs‐*epn*, we detected 4280, 902 and 1043 upregulated differently expressed genes (up‐DEGs), respectively (Figure 5A, Tables S2–, S5). Among these, 814 up‐DEGs were shared between *quintuple*‐vs‐WT and *octuple*‐vs‐WT (90.2% of *octuple*‐vs‐WT up‐DEGs), and 672 were common to all three comparisons (74.5% and 64.4% of *octuple*‐vs‐WT and *octuple*‐vs‐*epn* up‐DEGs, respectively) (Figure 5B). Gene ontology (GO) enrichment showed that the top terms for these shared up‐DEGs were predominantly immune‐related (Figure 5C–F, Tables S3–, S6).

Notably, the GO terms enriched among the up‐DEGs in the *octuple*‐vs‐WT and *octuple*‐vs‐*epn* comparisons included typical downstream immune pathways, such as “response to salicylic acid,” “defence response to bacterium,” “systemic acquired resistance,” “regulation of defence response,” “response to oxidative stress,” “response to bacterium,” and “response to jasmonic acid.” (Figure 5D,E, Tables S4 and S5). Moreover, the expression levels of these up‐DEGs were lower in the *octuple* mutant than in the *quintuple* mutant (Figure 5G, Table S2). These results suggest that blocking canonical ETI partially suppresses the hyperactive immunity, yet a substantial residual immune activation persists in *epn;quintuple*, independent of the classical pathway.

For down‐DEGs, we found 4367, 121 and 95 in the three comparisons, with only 37 common to all (Figure 5, Figure S7, Table S6). The GO enrichment terms of these down‐DEG sets differed among the three comparisons and were not immunity‐related (Figure S7). Moreover, the expression of the shared down‐DEGs was less reduced in *octuple* than in *quintuple* (Figure 5G and Table S2). These results suggest that these genes are unlikely to be the primary cause of developmental defects but rather secondary consequences. Among the 37 common down‐DEGs (Table S6), none corresponded to known mutations that cause plant autoimmunity or developmental abnormalities. The functions of several remain uncharacterized, and further investigation is needed to determine whether any of these genes contribute to constitutive immune activation or the *epn;quinple* phenotype.

### 2.6 CBP60g^D252N^ Suppresses the Autoimmunity of *cbp60s* Through a Pathway Independent of Canonical ETI

**FIGURE 6:**
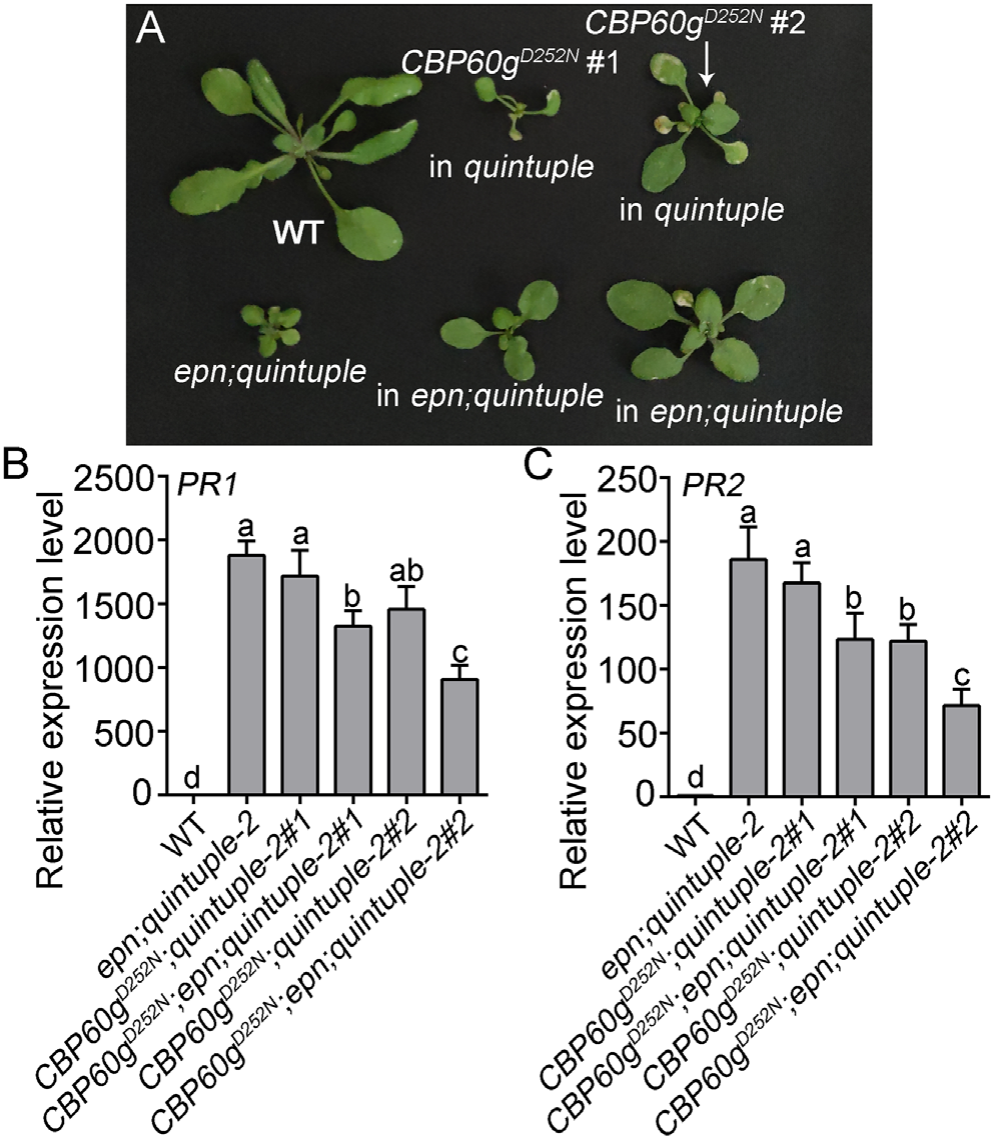
CBP60g^D252N^ partially rescues the autoimmunity in *epn;quintuple*. (A) Representative growth of wild‐type (WT), *epn;quintuple‐2* (*epn;quintuple*), *CBP60g* ^*D252N*^ *;quintuple‐2* #1, *CBP60g* ^*D252N*^ *;epn;quintuple‐2* #1, *CBP60g* ^*D252N*^ *;quintuple‐2* #2, *CBP60g* ^*D252N*^ *;epn;quintuple‐2* #2, at 3 weeks after germination (WAG) under long‐day (LD) conditions. (B, C) Relative transcript abundance of *PR1* (B) and *PR2* (C) in the indicated genotypes. RNAs were extracted from leaves of three WAG plants under LD conditions. Values are means ± SE (*n* = 4). Different letters indicate significantly different groups (one‐way ANOVA, Tukey's multiple comparisons test, *p* < 0.05).

These results indicate that both the introduction of CBP60g^D252N^ and the disruption of the canonical ETI pathway only partially suppress the *quintuple* phenotype. To investigate whether CBP60g^D252N^ suppresses the autoimmunity of *cbp60s* through the ETI pathway, we introduced CBP60g^D252N^ into the *epn;quintuple* background by crossing *epn;quintuple* with *UBQ10:CBP60g* ^*D252N*^ *;quintuple*. We found that CBP60g^D252N^ further complemented the developmental defects of *epn;quintuple* (Figure 6A) and further reduced the expression levels of the immune genes *PR1* and *PR2* (Figure 6B,C), suggesting that CBP60g^D252N^ suppresses the autoimmunity of the *quintuple* mutant through an as‐yet‐unknown pathway.

To further investigate whether high‐level expression of *CBP60g* ^*D252N*^ could completely suppress autoimmunity in the *quintuple* mutant, we introduced *UBQ10:CBP60g* ^*D252N*^ directly into the *epn;quintuple* background via floral dip transformation. From over 30 T_1_ transgenic lines, we selected two with the highest expression levels (Figure S8A). We observed that even at 5 weeks of growth, these lines still failed to bolt and set seeds (Figure S8B). Furthermore, we examined the expression of immune‐related genes in the *CBP60g* ^*D252N*^ *;epn;quintuple* lines and found that the transcript levels of key immune genes such as *SARD1*, *SID2*, *FMO1* and *BKK1* remained higher than those in wild‐type plants (Figure S8C–F). These results indicate that combining the introduction of CBP60g^D252N^ with disruption of the ETI pathway only partially suppresses autoimmunity in the *quintuple* mutant, and that immune responses remain constitutively activated in the CBP60g^D252N^ in *epn;quintuple*.

## 3 Discussion

In this study, we identified a novel *CBP60g* mutant allele, *cbp60g‐3*, through EMS mutagenesis screening. In the *cbp60g‐3* mutant, the amino acid D at position 252 of CBP60g is changed to N. This site is located in the TRM of the protein and may affect the binding of CBP60g to the subunits of the mediator complex or transcription complex (Yang et al. 2015; Chen et al. 2022). Although CBP60g^D252N^ exhibits reduced transcriptional activity compared to CBP60g (Figure 3), it retains the ability to complement resistance to the pathogen *P. syringae* pv. *tomato* DC3000 in the *cbp60g;sard1* mutant (Figure 3), indicating that this residue compromises its transcriptional activity but does not abolish its responsiveness to pathogens. Notably, this transcriptionally compromised CBP60g^D252N^ variant suppresses the *cbp60b* autoimmune phenotype (Figures 1 and 2), whereas wild‐type CBP60g does not (Figure 2) (Li et al. 2024). This indicates that the functional alteration of the CBP60g^D252N^, rather than a substitution for the transcriptional function of CBP60b, thereby suppresses the autoimmunity of *cbp60b*.

We demonstrate that autoimmunity in the *quintuple* mutant is mediated by both canonical ETI‐dependent and ‐independent pathways. Mutation of the CBP60b clade results in strong autoimmunity (Li et al. 2024), whereas disrupting the ETI pathway by introducing *eds1;pad4* and *ndr1* mutants only partially rescues the *quintuple* phenotype (Figure 4). Moreover, RNA‐seq data revealed that immune genes remained constitutively activated in the *epn;quintuple* mutant, with enrichment in typical downstream immune pathways, including SA, SAR and bacterial defence, as well as their regulatory components (Figure 5). These results suggest that additional ETI‐independent mechanisms contribute to the autoimmunity of the *quintuple* mutant. In support of the above results, introducing CBP60g^D252N^ further alleviated the *epn;quintuple* phenotype (Figure 6), yet immune gene expression remained substantially elevated and developmental defects persisted in the *CBP60g* ^*D252N*^ *;epn;quintuple* plants (Figure 6, Figure S8), indicating that both introduction of CBP60g^D252N^ and disruption of the canonical ETI pathway only partially suppress autoimmunity in the *quintuple* mutant.

Considering that the CBP60b clade emerged during plant colonization of land (Zheng et al. 2021; Li et al. 2024), they may play a role in plant development, and the functions of the downregulated genes in the *quintuple* and *epn;quintuple* mutants remain to be identified. Therefore, we cannot rule out the possibility that the phenotype of *epn;quintuple* results from developmental pathways regulated by the CBP60b clade, potentially involving a trade‐off between plant development and immunity.

The autoimmunity resulting from *CBP60b* mutation is distinctly different from that observed in other mutants. Although SA accumulation and SA/SAR pathways genes expression are elevated in the *cbp60b* mutant (Li et al. 2021), mutating components of the SA/SAR pathways did not suppress the *cbp60b* phenotype (Figure S1). In other autoimmune mutants caused by different gene types—such as the transcription factor mutant *camta1;2;3* (Sun et al. 2020), immune receptor mutants *snc1* (Zhang et al. 2003), *snc2‐1D* (Zhang, Yang, et al. 2010), and *bir1‐1* (Gao et al. 2009), as well as other types of mutants including *mpk4* (Brodersen et al. 2006), *syp121;syp122* (Zhang et al. 2008), *acd6‐1* (Lu et al. 2009), and *snap33* (Henchiri et al. 2024)—introducing *sid2* or *fmo1* mutant into these mutants' background suppresses the autoimmune phenotype to varying degrees. We mutated 11 *NLR* genes that are upregulated in *cbp60b*, but none of these mutations rescued the *cbp60b* phenotype (Figure S2).

However, mutations in EDS1‐PAD4 and NDR1 pathway fully or partially suppressed the *cbp60b* phenotype (Li et al. 2021), suggesting that both TNLs and CNLs may participate in the *cbp60b* autoimmunity. This raises several questions: (1) Why do SA and SAR pathways, as downstream components of EDS1‐PAD4, fail to suppress *cbp60b* autoimmunity? (2) If both classes of NLRs are involved in this process, why does disrupting the EDS1‐PAD4 pathway alone completely suppress *cbp60b* autoimmunity? Is there a mechanism in *cbp60b* that enables these two NLR classes to function cooperatively? The *quintuple* mutant exhibits severe autoimmunity, yet disrupting the ETI pathway does not fully suppress it, suggesting that mutations in CBP60c‐f may activate additional immune pathways—although *cbp60c;d;e;f* mutant only displays mild immune defects (Li et al. 2024). Furthermore, *cbp60g;sard1;cbp60b* displays autoimmunity comparable to the *quintuple* mutant, yet functional loss of the EDS1‐PAD4 pathway completely rescues the *cbp60g;sard1;cbp60b* phenotype (Li et al. 2021). These results indicate that the mechanism underlying *cbp60b* and *cbp60b;c;d;e;f* autoimmunity appears distinct from those reported for other known autoimmune mutants.

Although we have achieved some genetic insights, these findings imply that the CBP60 family members and the immune pathways they regulate are interconnected through complex and elaborate regulatory cascades rather than operating in a simply overlapping or discrete manner. This complexity renders the molecular mechanisms underlying the autoimmunity caused by their mutations challenging to unravel. At the same time, this presents a fascinating scientific question. Future efforts to isolate additional suppressors through EMS mutagenesis may help identify the immune components involved in this process, gradually unravelling the underlying mechanisms.

## 4 Experimental Procedures

### 4.1 Plant Materials and Growth Conditions

*Arabidopsis thaliana* Columbia‐0 was used as the wild type. Plant materials including *cbp60b‐1* (SAIL_40_E09), *cbp60b‐2* (GK‐521D01‐020205), *sard1;cbp60g*, *quintuple‐1*, *quintuple‐2*, *eds1‐c1*, *pad4‐c1*, *ndr1‐c1* and *CBP60b‐f genomic:GUS* reporter lines were described previously (Li et al. 2021, 2024). Mutants, including *fmo1‐c1*, *fmo1‐c2*, *sid2‐c1*, *sid2‐c2*, *rmg1‐c1*, *tn2‐c1*, *soc3‐c1*, *at5g38340‐c1*, *at3g04220‐c1*, *at5g46260‐c1*, *at5g66630‐c1*, *at1g58390‐c1*, *at1g12210‐c1*, *at5g18350‐c1* and *at1g64070‐c1*, were generated using CRISPR‐Cas9 as described (Wang et al. 2015). Surface‐sterilized seeds were plated on half‐strength Murashige and Skoog (1/2 MS) basal medium plates, kept at 4°C in darkness for 4 days, then transferred to a growth chamber at 22°C for germination, and later transplanted to soil. Plants were grown in a growth chamber at 22°C under long‐day (LD) condition (16 h light/8 h dark) or short‐day (SD) condition (12 h light/12 h dark). Stable *Arabidopsis* transformations were performed using the floral dipping method. Transgenic plants were selected on 1/2 MS medium supplemented with 30 μg/mL Basta salt (Sigma‐Aldrich) or 25 mg/mL hygromycin (Roche).

### 4.2 Plasmid Construction

Constructs were generated using the Gateway technology (Invitrogen) unless noted otherwise. The pENTR/D/TOPO vector (Invitrogen) was used to generate entry vectors. The entry vectors for the coding sequences of *CBP60g*, CBP60a, SARD1 and bHLH^MYC2^‐CBP60g‐TRM were described previously (Li et al. 2021, 2024). PCR fragments of *NLR DN* were amplified by using primer pairs, W53/W54/W55/W56 for *RMG1 DN*, W49/W50/W51/W52 for *TN2 DN*, W57/W58/W59/W60 for *AT3G04220 DN*, W101/W102/W105/W106 for *AT5G38340 DN*, W107/W108/W111/W112 for *AT1G58390 DN*, and then were inserted into the entry vector. The mutational entry vectors were generated with primers, W1481a/W1482a for *CBP60g* ^*D252N*^ and *bHLH* ^*MYC2*^ *‐CBP60g* ^*D252N*^ *‐TRM*, W1649/W1650 for *SARD1* ^*D259N*^, W1736/W1737 for *CBP60a* ^*D241N*^, using pEASY‐Uni Seamless Cloning and Assembly Kit (Transgen Biotech).

Expression vectors for dual luciferase reporter assays and transgenic plants were as followed. Expression constructs *35S:bHLH* ^*MYC2*^ *–GFP*, 3*5S:bHLH* ^*MYC2*^ *‐CBP60g‐TRM–GFP*, and LUC reporter constructs *pTPS10:LUC*, pEDS1:LUC, and pSID2:LUC were described previously (Li et al. 2021). Entry vectors were used in LR reactions with the destination vector *UBQ10:GW‐GFP* or *35S:GW‐GFP* (Li et al. 2021) to generate *UBQ10:CBP60g‐GFP*, *UBQ10:CBP60g* ^*D252N*^ *‐GFP*, 3*5S:bHLH* ^*MYC2*^ *‐CBP60g‐TRM–GFP*, *UBQ10:CBP60a* ^*D241N*^ *‐GFP*, and *UBQ10:SARD1* ^*D259N*^ *‐GFP*.

CRISPR‐Cas9 constructs were generated with gene‐specific sequences amplified with corresponding primers (Table S1) and with *pCBC‐DT1T2* as the template (Xing et al. 2014). PCR fragments were inserted into *pHEE401E* using restriction‐ligation reactions as described (Wang et al. 2015). Sequencing with target‐specific primers (Table S1) was performed to verify genomic editing of the targets.

All PCR amplifications used Phusion hot start high‐fidelity DNA polymerase at the annealing temperature and extension times recommended by the manufacturer. The Bioneer PCR purification kit and Bioneer Spin miniprep kit were used for PCR product recovery and plasmid DNA extraction, respectively. All constructs were sequenced and analysed using Vector NTI. Primers are listed in Table S1.

### 4.3 RT‐PCRs and RT‐qPCRs

Total RNAs were extracted from the 5th to 6th true leaves of 2 or 3 WAG plants under LD condition or 5 WAG plants under SD condition except where noted. All experiments were repeated in three to four biological replicates. Each biological replicate included 8–10 leaves from five plants. Total RNAs were isolated using Ultrapure RNA Kit (Cwbiotech) according to the instructions of manufacturers. Reverse transcription was performed with ReverTra Ace qPCR RT Master Mix with gDNA Remover (Toyobo). RT‐qPCRs were performed with the ABI QuantStudio 6 Flex using SYBR Green real‐time PCR master mix (Toyobo). *GAPDH* was used as a quantitative control for RT‐qPCRs. The relative expression of genes was analysed by the 2^−∆∆*C*t^ method (Livak and Schmittgen 2001). All primers are listed in Table S1.

### 4.4 RNA‐Sequencing and Data Analysis

For RNA‐sequencing, aerial parts of 2‐week‐old *Arabidopsis* seedlings under LD condition were collected. The 20–30 seedlings mixing pool was collected and then frozen in liquid nitrogen, followed by RNA extraction. Three independent replicates were performed.

Total RNA was extracted using the TRIzol reagent (Invitrogen) according to the manufacturer's protocol. The libraries were sequenced on an illumina Novaseq 6000 platform at Oebiotech. About 50 million raw reads for each sample were generated, and the raw data have been submitted to the NCBI Gene Expression Omnibus (GEO) datasets with accession number GSE335760. Differential expression analysis was performed using DESeq2. *q*‐value < 0.05 and fold change > 2 or fold change < 0.5 were set as the thresholds for significant DEGs. GO enrichment analysis of DEGs were performed to screen the significant enriched term using R (v. 3.2.0) on the OECloud platform.

### 4.5 Pathogen Inoculation and Bacterial Growth Assays

*Pseudomonas syringea* pv. *tomato* DC3000 was cultured at room temperature (RT) in King's B medium (protease peptone, 10 mg/mL; glycerol, 15 mg/mL; K_2_HPO_4_, 1.5 mg/mL; MgSO_4_, 5 mM, pH 7.0) supplemented with 12.5 mg/mL rifampicin. Plants at 5 WAG under SD condition were used for inoculation. DC3000 suspensions in 10 mM MgCl_2_ (OD_600_ = 0.0001) were infiltrated into the abaxial sides of mature leaves using a needleless 1‐mL syringe. To determine the bacterial titres (CFU/cm^2^), leaves were sampled at 2 h and 3 days by 0.5 cm diameter puncher. Samples were ground, serially diluted tenfold, and plated on King's B medium plates. The number of colonies was counted after 2 days of culture at RT.

### 4.6 Dual LUC Reporter Assays in *Arabidopsis* Protoplasts

Leaves of 3 WAG *Arabidopsis* plants grown under 16 h light/8 h dark were harvested and protoplasts were isolated following the tape‐*Arabidopsis* sandwich method (Wu et al. 2009). Protoplasts were co‐transformed with various combinations of constructs using the polyethylene glycol/calcium‐mediated method (Yoo et al. 2007). LUC activity was tested with a Double‐LUC Reporter Assay Kit (TransGen Biotech) using the Dual‐Light Chemiluminescent Reporter Gene Assay System (Berthold). The ratio of LUC driven by the promoters to Renilla LUC driven by the CaMV 35S promoter was calculated to determine the transcriptional activities, as described (Li et al. 2024). All experiments were repeated in three to four biological replicates. Different letters indicate significantly different groups (one‐way ANOVA, Tukey's multiple comparisons test, *p* < 0.05).

### 4.7 EMS Mutagenesis

About 10,000 *cbp60b‐1* seeds were incubated with 25 mL of 0.2% (v/v) EMS in a 50‐mL Falcon tube on a tube rotator for 15 h. The seeds were rinsed with water 10 times before being dried under a fume cupboard. A total of 1000 M_2_ progeny derived from M_1_ plants were analysed, and *sw‐05‐12* was selected based on their growth conditions. After two rounds of backcrossing with *cbp60b/+*, the material was sent to a sequencing company (Oebiotech) for whole‐genome resequencing, and the mutation was ultimately mapped to the *CBP60g* gene.

### 4.8 Histochemical GUS Staining

The 5 DAG seedlings with GUS reporter were stained with X‐Gluc (Sigma‐Aldrich) substrates at 37°C for 8 h. Then, the seedlings were transferred to 70% ethanol to remove the residual colour and captured by an Olympus BX51 microscope.

## Author Contributions

**Sha Li:** funding acquisition, writing – original draft, supervision. **Shu‐Lei Wang:** funding acquisition, project administration. **Lu‐Shen Li:** conceptualization, validation, funding acquisition, writing – original draft, writing – review and editing, project administration, supervision, data curation, investigation, formal analysis, visualization, resources, methodology. **Lin Ma:** investigation, software, data curation, validation. **Yan‐Yan Yang:** investigation.

## Funding

This work is funded by National Natural Science Foundation of China (32500247 to Lu‐Shen Li and 32400282 to Shu‐Lei Wang), Shandong Provincial Natural Science Foundation (ZR2024MC093 to Sha Li).

## Conflicts of Interest

The authors declare no conflicts of interest.

## Supporting information


**Figure S1:** Functional loss of classical salicylic acid (SA) and systemic acquired resistance (SAR) pathways fail to rescue the defects in *cbp60b*.

**Figure S2:** Functional loss of upregulated *NLRs* in *cbp60b* fail to rescue the defects in *cbp60b*.

**Figure S3:** Expression analysis of endogenous, exogenous, and total *CBP60g* transcripts.

**Figure S4:** CBP60g^D252N^ partially rescues the defects in *quintuple*.

**Figure S5:** Functional loss of canonical effector‐triggered immunity (ETI) pathways only partially rescue the autoimmunity in *quintuple*.

**Figure S6:** The expression pattern of *CBP60b‐f* in aerial tissues.

**Figure S7:** Gene ontology analysis of downregulated differentially expressed genes (DEGs) in indicated groups.

**Figure S8:** Overexpression of CBP60g^D252N^ cannot completely rescue the autoimmunity in *epn;quintuple*.

**Table S1:** Oligonucleotides used in this study.

**Table S2:** DEG_combinded_nofiltered.

**Table S3:** Quintuple‐vs‐WT DGE and GO top.

**Table S4:** octuple‐vs‐WT DGE and GO top.

**Table S5:** Octuple‐vs‐triple DGE and GO top.

**Table S6:** Common DGE and GO top.

## Data Availability

The data that support the findings of this study are available from the corresponding author upon reasonable request. *Arabidopsis* genes involved can be found in TAIR under the following accession numbers: AT5G62570 for *CBP60a*, AT5G57580 for *CBP60b*, AT2G18750 for *CBP60c*, AT4G25800 for *CBP60d*, AT2G24300 for *CBP60e*, AT4G31000 for *CBP60f*, AT5G26920 for *CBP60g*, AT1G73805 for *SARD1*, AT1G74710 for *SID2*, AT2G14610 for *PR1*, AT3G57260 for *PR2*, AT1G32640 for *MYC2*, AT2G24210 for *TPS10*, AT3G04120 for *GAPDH*. AT3G48090 for *EDS1*, AT3G52430 for *PAD4*, AT3G20600 for *NDR1*, AT1G19250 for *FMO1*, AT4G11170 for *RMG1*, AT1G17615 for *TN2*, AT1G17600 for *SOC3*, AT2G13790 for *BKK1*/*SERK4*.
