# Supplementary material for: Canonical ETI‐Dependent and ‐Independent Pathways Mediate Autoimmunity Caused by Loss of CBP60b Clade Function
Source: Mol Plant Pathol. 2026 Jul 11;27(7):e70318. doi: 10.1111/mpp.70318 (PMC13354941; doi:10.1111/mpp.70318)
Supplement: Supplementary file 1 — Figure S1: Functional loss of classical salicylic acid (SA) and systemic acquired resistance (SAR) pathways fail to rescue the defects in cbp60b. [file MPP-27-e70318-s014.docx]

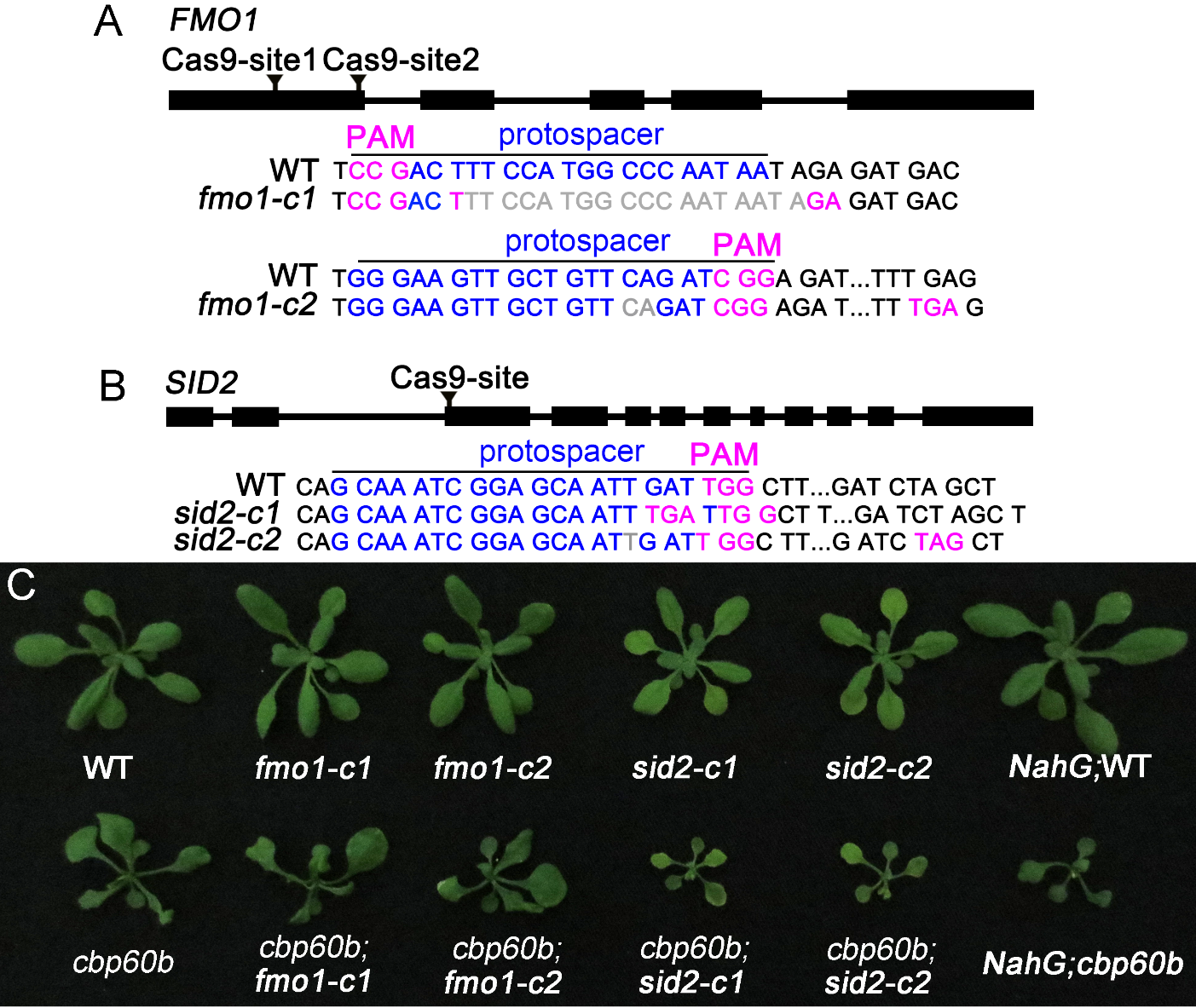


**Supplemental Figure 1. Functional loss of classical SA and SAR pathways fail to rescue the defects in *cbp60b*.**

(A-B) Genomic structure of *FMO1* (A), and *SID2* (B). Target sites by Cas9 were indicated by inverted triangles on the genomic loci. Cas9-generated base pair deletions or insertions are indicated by grey or magenta letters, respectively. (C) Representative growth of WT, *cbp60b-1* (*cbp60b*), *fmo1-c1*, *fmo1-c2*, *sid2-c1*, *sid2-c2*, *cbp60b;fmo1-c1*, *cbp60b; fmo1-c2*, *cbp60b; sid2-c1*, *cbp60b; sid2-c2*, *NahG* in WT, and *NahG* in *cbp60b* at 3 WAG under LD conditions.
