# Supplementary material for: Canonical ETI‐Dependent and ‐Independent Pathways Mediate Autoimmunity Caused by Loss of CBP60b Clade Function
Source: Mol Plant Pathol. 2026 Jul 11;27(7):e70318. doi: 10.1111/mpp.70318 (PMC13354941; doi:10.1111/mpp.70318)
Supplement: Supplementary file 2 — Figure S2: Functional loss of upregulated NLRs in cbp60b fail to rescue the defects in cbp60b. [file MPP-27-e70318-s005.docx]

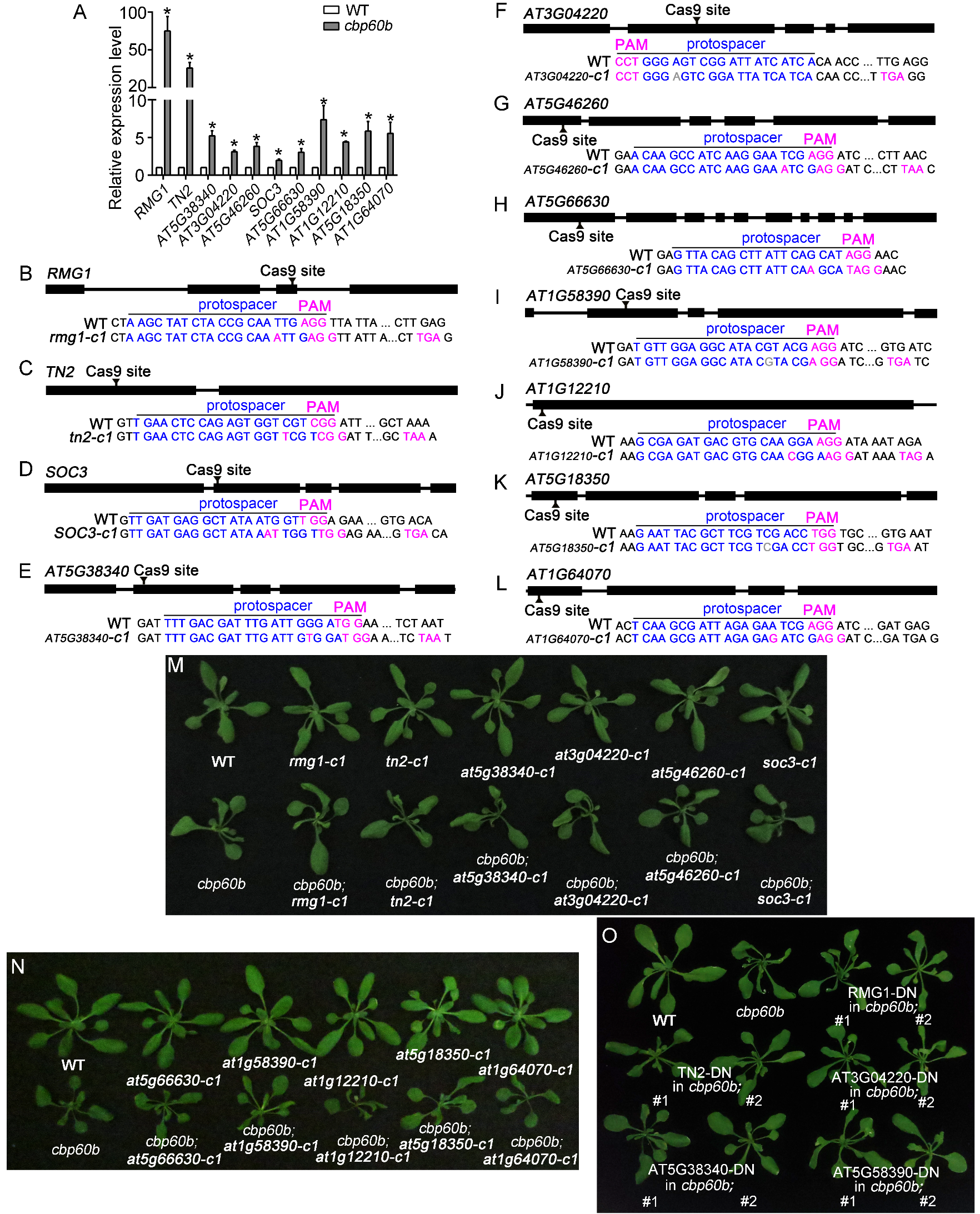


**Supplemental Figure 2. Functional loss of up-regulated *NLRs* in *cbp60b* fail to rescue the defects in *cbp60b*.**

(A) Relative transcript abundance of *NLRs* in WT and *cbp60b* by RT‐qPCRs. Asterisks indicate significant difference (t test; *P* < 0.05) (B-L) Genomic structure of *RMG1* (B), *TN2* (C), *SOC3* (D), *AT5G38340* (E), *AT3G04220* (F), *AT5G46260* (G), *AT5G66630* (H), *AT1G58390* (I), *AT1G12210* (J), *AT5G18350* (K) and *AT1G64070* (L). Target sites by Cas9 were indicated by inverted triangles on the genomic loci. Cas9-generated base pair deletions or insertions are indicated by grey or magenta letters, respectively. (M-O) Representative growth of the indicated genotypes at 3 WAG under LD conditions.
