# Supplementary material for: Canonical ETI‐Dependent and ‐Independent Pathways Mediate Autoimmunity Caused by Loss of CBP60b Clade Function
Source: Mol Plant Pathol. 2026 Jul 11;27(7):e70318. doi: 10.1111/mpp.70318 (PMC13354941; doi:10.1111/mpp.70318)
Supplement: Supplementary file 3 — Figure S3: Expression analysis of endogenous, exogenous, and total CBP60g transcripts. [file MPP-27-e70318-s002.docx]

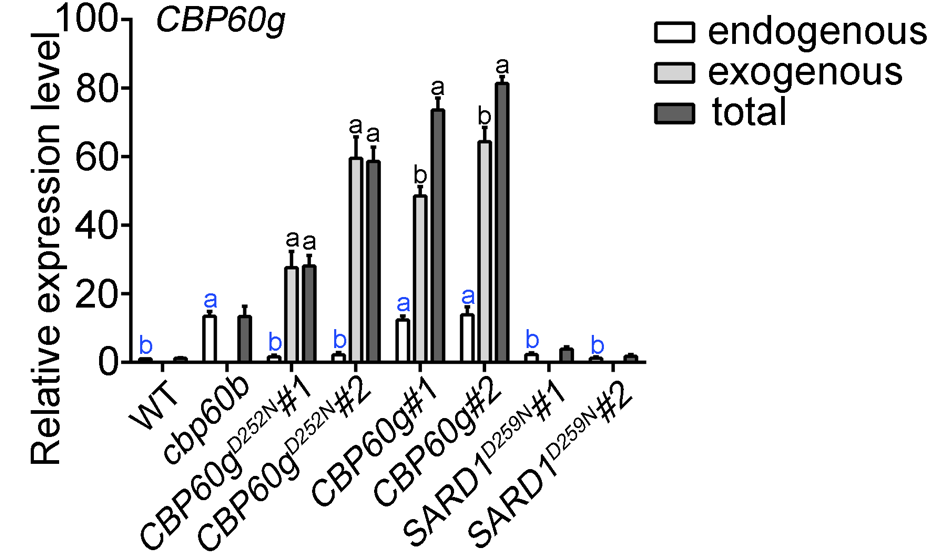


**Supplemental Figure 3. Expression analysis of endogenous, exogenous, and total CBP60g *transcripts.***

(A) Relative transcript abundance of endogenous, exogenous, and total *CBP60g* transcripts in the indicated genotypes by RT‐qPCRs. Values are means ± SE (n=3). Different letters indicate significantly different groups (1-Way ANOVA, Tukey’s multiple comparisons test, *P*<0.05). Blue letters indicate statistical significance of endogenous *CBP60g* transcript levels among different genotypes, whereas black letters indicate statistical significance between exogenous and total transcripts within the same genotype.
