# Supplementary material for: Canonical ETI‐Dependent and ‐Independent Pathways Mediate Autoimmunity Caused by Loss of CBP60b Clade Function
Source: Mol Plant Pathol. 2026 Jul 11;27(7):e70318. doi: 10.1111/mpp.70318 (PMC13354941; doi:10.1111/mpp.70318)
Supplement: Supplementary file 4 — Figure S4: CBP60gD252N partially rescues the defects in quintuple. [file MPP-27-e70318-s011.docx]

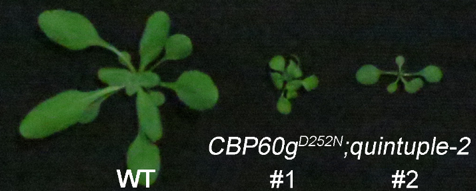


**Supplemental Figure 4. CBP60g^D252N^ partially rescues the defects in *quintuple*.**

Representative growth of WT and 2 independent lines of *UBQ10:CBP60g^D252N^-GFP;quintuple-2* at 3 WAG under LD conditions. Note that the *quintuple* plants die around 1-2 WAG and thus a representative plant is not included in the image taken at 3 WAG.
