# Supplementary material for: Canonical ETI‐Dependent and ‐Independent Pathways Mediate Autoimmunity Caused by Loss of CBP60b Clade Function
Source: Mol Plant Pathol. 2026 Jul 11;27(7):e70318. doi: 10.1111/mpp.70318 (PMC13354941; doi:10.1111/mpp.70318)
Supplement: Supplementary file 5 — Figure S5: Functional loss of canonical effector‐triggered immunity (ETI) pathways only partially rescue the autoimmunity in quintuple. [file MPP-27-e70318-s013.docx]

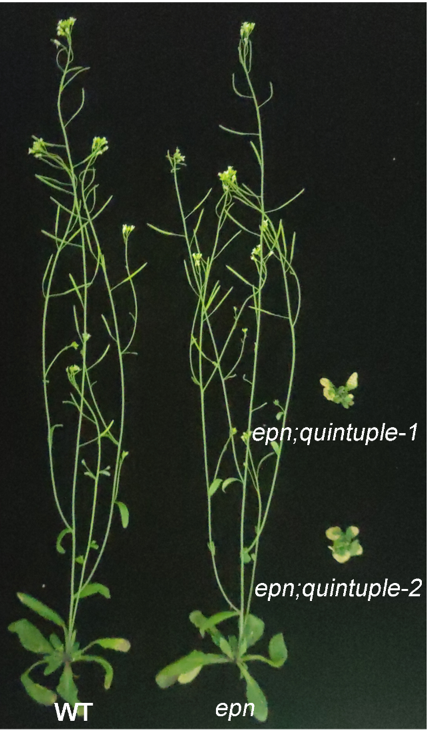


**Supplemental Figure 5. F****unctional loss of canonical ETI pathways only** **partially rescue the autoimmunity in *quintuple*.**

Representative growth of WT, *epn*, *epn;quintuple-1*, and *epn;quintuple-2* at 5 WAG under LD conditions. Note that the *quintuple* plants die around 1-2 WAG and thus a representative plant is not included in the image taken at 5 WAG.
