# Supplementary material for: Canonical ETI‐Dependent and ‐Independent Pathways Mediate Autoimmunity Caused by Loss of CBP60b Clade Function
Source: Mol Plant Pathol. 2026 Jul 11;27(7):e70318. doi: 10.1111/mpp.70318 (PMC13354941; doi:10.1111/mpp.70318)
Supplement: Supplementary file 6 — Figure S6: The expression pattern of CBP60b‐f in aerial tissues. [file MPP-27-e70318-s004.docx]

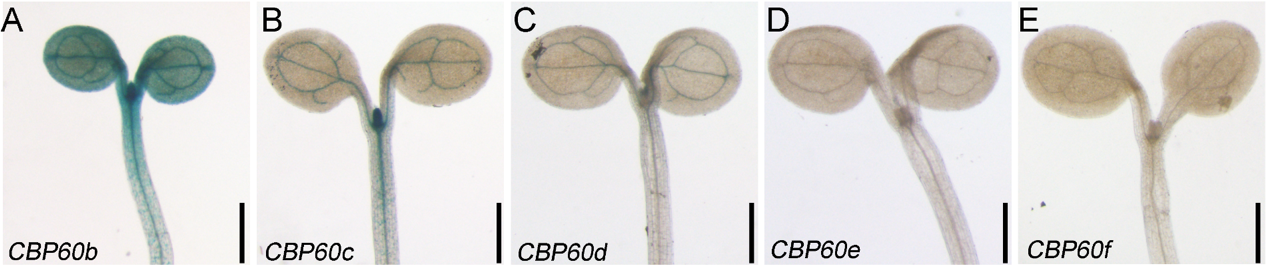


**Supplemental Figure 6. The expression pattern of *CBP60b-f* in aerial tissues.**

(A-E) Expression of *CBP60b-f* by histochemical GUS staining of *genomic:GUS* reporter lines at 5 DAG under LD conditions. Bars = 1 mm
