# Supplementary material for: Canonical ETI‐Dependent and ‐Independent Pathways Mediate Autoimmunity Caused by Loss of CBP60b Clade Function
Source: Mol Plant Pathol. 2026 Jul 11;27(7):e70318. doi: 10.1111/mpp.70318 (PMC13354941; doi:10.1111/mpp.70318)
Supplement: Supplementary file 7 — Figure S7: Gene ontology analysis of downregulated differentially expressed genes (DEGs) in indicated groups. [file MPP-27-e70318-s003.docx]

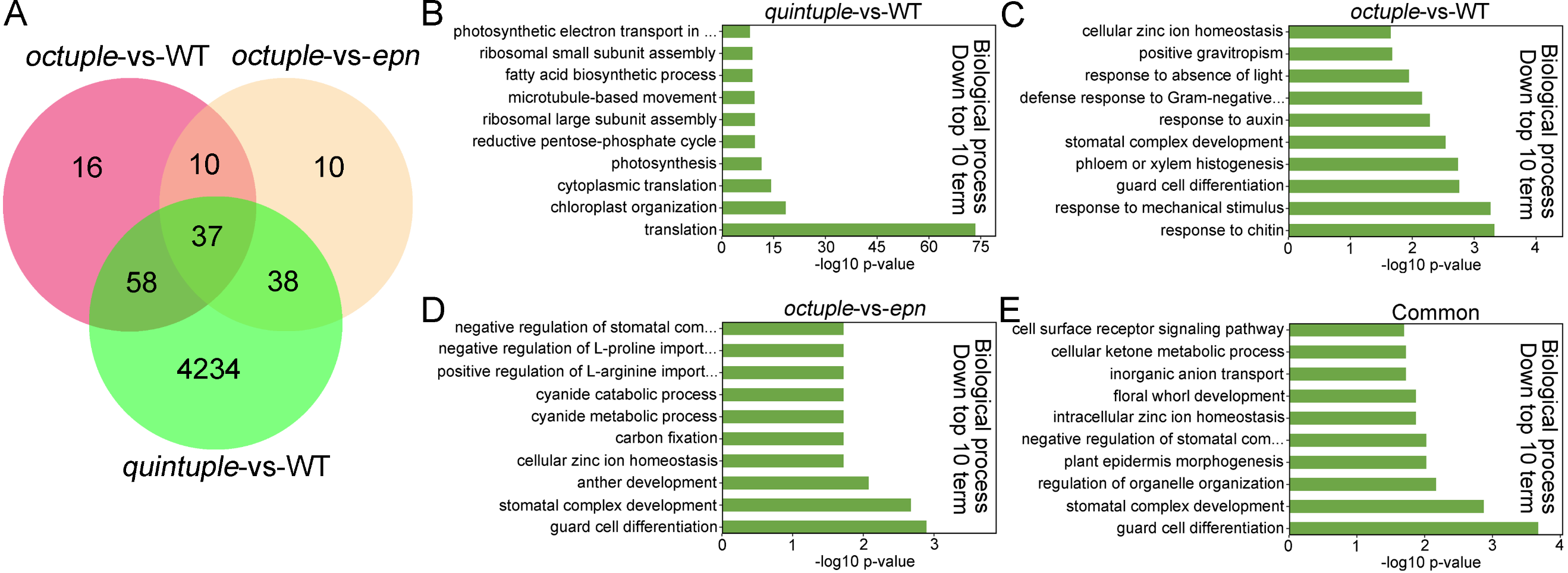


**Supplemental Figure 7. Gene ontology analysis of down-DEGs in indicated groups.**

(A) Venn diagram shows the overlapping numbers of down-DEGs in indicated groups. (B-E) Gene ontology enrichment of down-DEGs in *quintuple*-vs-WT (B), *octuple*-vs-WT (C) and *octuple*-vs-*epn* (D), and common down-DEGs (E). The lengths of the bars indicate the -log10-transformed p-values.
