# Supplementary material for: Canonical ETI‐Dependent and ‐Independent Pathways Mediate Autoimmunity Caused by Loss of CBP60b Clade Function
Source: Mol Plant Pathol. 2026 Jul 11;27(7):e70318. doi: 10.1111/mpp.70318 (PMC13354941; doi:10.1111/mpp.70318)
Supplement: Supplementary file 8 — Figure S8: Overexpression of CBP60gD252N cannot completely rescue the autoimmunity in epn;quintuple. [file MPP-27-e70318-s007.docx]

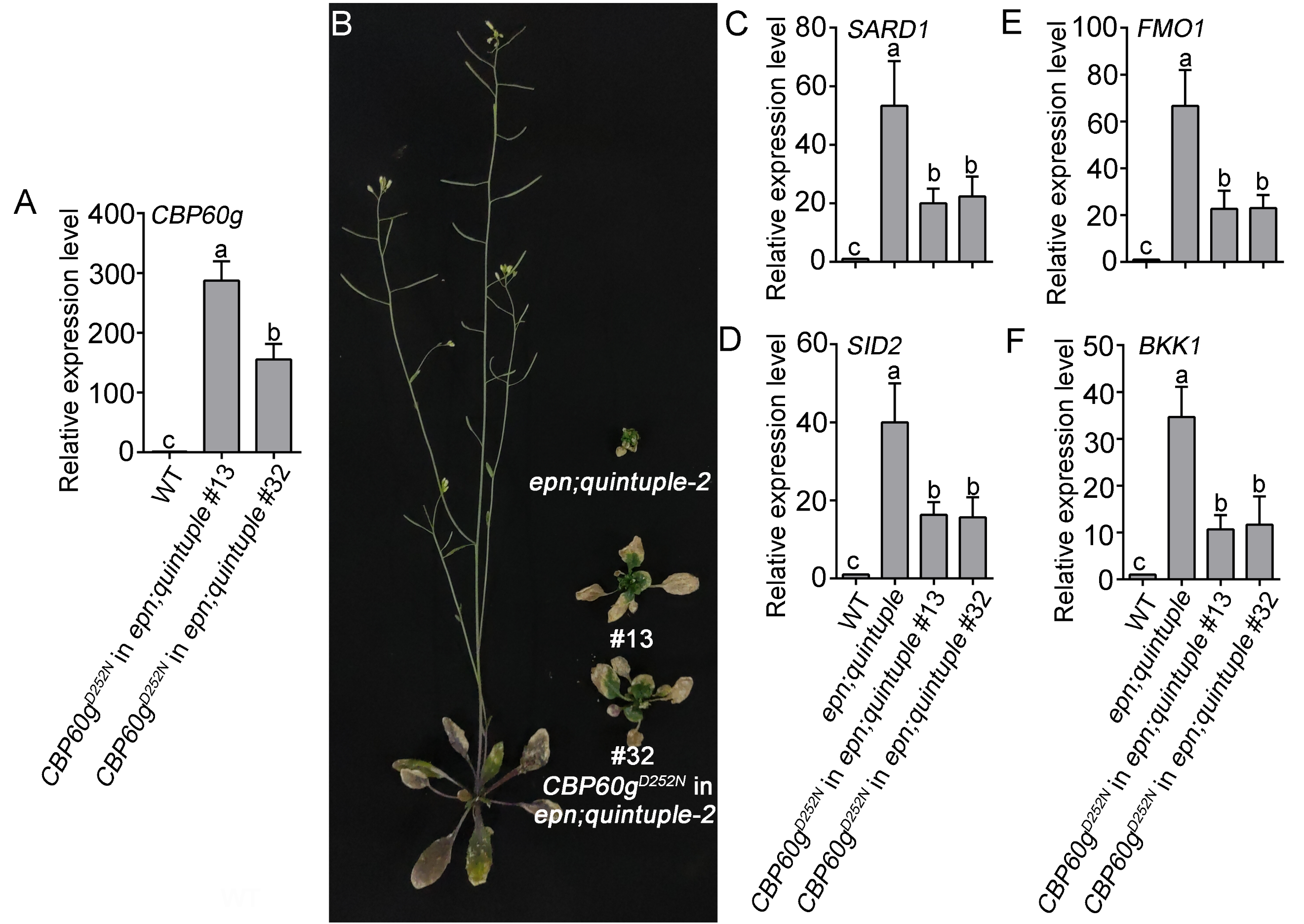


**Supplemental Figure 8.** **Overexpression of CBP60g^D252N^ cannot completely rescue the autoimmunity in *epn;quintuple.***

(A) Relative transcript abundance of *CBP60g* or *CBP60g^D252N^* in WT or 2 independent lines of *UBQ10:CBP60g^D252N^‐GFP* by RT‐qPCRs. (B) Representative growth of WT, *epn;quintuple-2*, *CBP60g^D252N^* in *epn;quintuple-2* #13 and *CBP60g^D252N^* in *epn;quintuple-2* #32 at 5 WAG under LD conditions. (C-F) Relative transcript abundance of *SARD1* (C), *SID2* (D), *FMO1* (E), and *BKK1* (F) in the indicated genotypes. RNAs were extracted from leaves of 3 WAG plants under LD condition. Values are means ± SE (n=4). Different letters indicate significantly different groups (1-Way ANOVA, Tukey’s multiple comparisons test, *P*<0.05).
